# Systematic Review of Reflection Spectroscopy-Based Skin Carotenoid Assessment in Children

**DOI:** 10.3390/nu15061315

**Published:** 2023-03-07

**Authors:** Saima Hasnin, Dipti A. Dev, Taren Swindle, Susan B. Sisson, Stephanie Jilcott Pitts, Tirna Purkait, Shari C. Clifton, Jocelyn Dixon, Virginia C. Stage

**Affiliations:** 1 Department of Child, Youth and Family Studies, University of Nebraska-Lincoln, Lincoln, NE 68588, USA; 2 Department of Family and Preventive Medicine, University of Arkansas for Medical Sciences, Little Rock, AR 72205, USA; 3 Department of Allied Health Sciences, University of Oklahoma Health Sciences Center, Oklahoma City, OK 73126, USA; 4 Department of Public Health at the Brody School of Medicine, East Carolina University, Greenville, NC 27834, USA; 5 Department of Nutrition and Health Sciences, University of Nebraska-Lincoln, Lincoln, NE 68588, USA; 6 Robert M. Bird Health Sciences Library, University of Oklahoma Health Sciences Center, Oklahoma City, OK 73126, USA; 7 Department of Human Development and Family Science, East Carolina University, Greenville, NC 27834, USA; 8 Department of Agricultural and Human Sciences, North Carolina State University, Raleigh, NC 27695, USA

**Keywords:** reflection spectroscopy, Veggie Meter, skin carotenoid score, fruit and vegetable consumption, meta-analyses, validity, children

## Abstract

Assessing children’s skin carotenoid score (SCS) using reflection spectroscopy (RS) is a non-invasive, widely used method to approximate fruit and vegetable consumption (FVC). The aims for the current review were to (1) identify distributions of SCS across demographic groups, (2) identify potential non-dietary correlates for RS-based SCS, (3) summarize the validity and reliability of RS-based SCS assessment, and (4) conduct meta-analyses of studies examining the correlation between RS-based SCS with FVC. A literature search in eight databases in June 2021 resulted in 4880 citations and peer-reviewed publications written in English that investigated children’s (2–10 years old) SCS using RS. We included 11 studies (intervention = 3, observational = 8). Potential covariates included weight status, ethnicity, seasonal variation, age, sex, and income. Studies reported criterion validity with children’s FVC but not with plasma carotenoid. Additionally, no studies reported the reliability of RS-based SCS in children. Among the 726 children included in the meta-analysis, the correlation between RS-based SCS and FVC was *r* = 0.2 (*p* < 0.0001). RS-based SCS is a valid method to quantify skin carotenoids for children’s FVC estimation with the potential for evaluating nutrition policies and interventions. However, future research should use standardized protocol for using RS and establish how RS-based SCS can translate to the amount of daily FVC in children.

## 1. Introduction

Daily fruit and vegetable consumption (FVC) has important protective effects against obesity and associated chronic conditions [1,2]. Despite the benefits of FVC, very few children consume the recommended amounts of fruits and vegetables [3,4,5]. For example, in the United States (U.S.), 88% of children do not meet the recommendations for vegetable intake and 50% fall short of the recommendations for whole fruit consumption [6]. Therefore, in recent years, increasing children’s FVC has been a common primary objective for public health nutrition interventions [7,8]. However, to assess the effectiveness of interventions designed to increase children’s FVC, accurate measures of FVC in children are needed. Current methods of measuring FVC in children between 2 and 10 years old are dependent on proxy reporting by caregivers [9,10,11], which is fraught with several sources of error, such as parents’ perceptions of their children’s preferences [12,13], recall bias, variable levels of literacy and numeracy, social desirability bias, and cognitive abilities [11]. Moreover, many U.S. children consume at least one meal and two snacks away from home [14], which makes it more difficult to collect reliable dietary intake data via parent report. Other validated methods to collect child-level dietary data include direct observations [15], plate waste [16], and remote food photography [17], all of which are beset with error and high expense [11]. Taken together, FVC data collection among children is time- and labor-intensive for researchers and study participants alike. Consequently, there is a critical need for an objective, unbiased, and non-invasive assessment of children’s FVC.

Carotenoids are the colorful substances in fruits and vegetables and they have many health benefits in and of themselves [18,19]. Fruits and vegetables are the predominant sources of carotenoids in the human diet [20]. While plasma carotenoids are the criterion standard for FVC approximation [21], skin carotenoid (SC) assessment using resonance Raman spectroscopy (RRS) is a comparatively newer method for the estimation of FVC in children [22,23,24].

RRS is a valid, non-invasive, objective, and reliable technology to assess SCS in children to estimate FVC [23,25,26,27,28]. RRS uses a narrow beam of light to excite the SC molecules leading to an enhanced reflection of Raman scattering. The device uses an algorithm to calculate the SC concentration, which is proportional to the number of reflected photons [29,30]. Previous studies validated the RRS method to measure SCS in children and adults using the NuSkin S3 Biophotonic Scanner device [31]. However, this method requires expensive instrumentation and analysis software, and the instrument is only available upon leasing from a proprietary company or is custom-built [31].

A newer and advanced alternative method to RRS for SC assessment includes pressure-mediated reflection spectroscopy (RS) [32,33,34]. RS has been validated with plasma carotenoid status in adults [32,34,35,36] but not in children. RS uses a broader band of LED light from 350 to 850 nm of the spectral range [33]. The portable RS device adds supradermal pressure of about 1 atmospheric pressure unit during the measurement at the illuminated spot of the skin. This pressure helps to temporarily reduce blood flow, which minimizes the interference of other biological pigments, thus enhancing the efficiency of RS over that of RRS to detect skin carotenoids [37]. RS uses a spectral de-convoluted algorithm to account for residual deoxyhemoglobin levels along with static skin melanin absorption levels in the calculation of the skin carotenoid absorption strength [33]. To date, the research-grade RS-based device available for purchase is the Veggie Meter^®^ (VM^®^) by the Longevity Link Corporation [38,38]. The VM^®^ has been established as a valid tool for SCS assessment in adults [34], and has been validated against plasma carotenoid concentrations [39] and RRS [32], which is considered a valid tool to measure SCS. Additionally, for the VM^®^, the correlation between SCS and plasma carotenoid ranges from 0.54 to 0.87 in adults [34,40,41], and the correlation between VM^®^-measured SCS and self-reported FVC was 0.69 in adults in one study [40]. Thus, due to its availability and lower cost than RRS, RS has emerged as a valid method to measure SCS to approximate FVC among adults. 

While RS-based SCS has been validated for use in adults, there is limited information on RS-based SCS validation in children. Specifically, Radtke et al. (2020) [41] reviewed spectroscopy-based FVC assessment methods in peer-reviewed literature published until 2018, finding that RRS was generally validated, yet only two studies used RS to assess SCS and neither of these two studies were among children. Given the research and policy emphasis on preventing childhood obesity and diet-related diseases [42], RS is increasingly being used among researchers and program evaluators. Therefore, the current systematic review examined the use of RS in children aged 2 to10 years old, and had the following aims:

(1) To identify distributions of SCS across demographic groups (age, biological sex, race, and ethnicity);
(2) To identify potential non-dietary correlates for RS-based SCS;
(3) To summarize the validity and reliability of RS-based SCS assessment in children as a proxy for FVC;
(4) To conduct a meta-analysis of studies examining the correlation between RS-based SCS and self-reported FVC in children.

Finally, we used the findings of this review to discuss implications for future research with RS-based SCS assessment in children.

## 2. Materials and Methods

### 2.1. Protocol Registration

We registered the current systematic review in the International Prospective Register of Systematic Reviews (PROSPERO) (CRD42021247220), though at the time of submission of this paper, the PROSPERO team had not reviewed it due to heightened focus given towards the COVID-19-related projects. For conducting this review and reporting, we followed the PRISMA (Preferred Reporting Items for Systematic Reviews and Meta-Analyses) statement [43].

### 2.2. Literature Search Strategy

The research team conducted a comprehensive literature search in eight search databases, which included PubMed, Scopus, Web of Science, Medline, Excerpta Medica Database (EMBASE), Cumulative Index of Nursing and Allied Health Literature (CINAHL), Cochrane Database of Systematic Reviews (CDSR), and Cochrane Central Register of Controlled Trials (CENTRAL). One co-author (S.C.C.), a research librarian, conducted the search in multiple databases for literature addressing the concepts of spectrum analysis, spectroscopy, and related terms; skin or blood; and carotenoids. S.C.C. conducted the initial search in May 2021, and later updated the search in February 2021 to retrieve any articles published after the initial search. Results included research articles published between January 1990 and February 2022 to capture field use of the RS for assessing SCS in children aged 2 to 10 years old. Appendix A includes the strategy from Ovid MEDLINE detailing the controlled vocabulary terms, keywords, and special features (e.g., limits, explode, focus, etc.) utilized in the search. Additionally, researchers conducted manual searches using the reference lists of the relevant articles. The first author (S.H.) manually searched references from the included articles and relevant systematic reviews (e.g., Radtke et al. 2020 [41]) to ensure no related articles were missed in the initial database searches. After downloading the search results, S.H. imported the references into the Mendeley citation manager to identify and remove duplicate citations. Then, S.H. imported unique items into DistillerSR (Evidence Partners, Ottawa, Canada) for title and abstract screening.

### 2.3. Data Collection and Analysis 

#### Selection of Studies

The research team used a structured form with four questions based on the inclusion criteria with three response options: yes, no, and cannot be determined. The four inclusion criteria for the title and abstract screening were (a) participants included children aged 2–10 years old; (b) the study assessed participants’ SCS; (c) the article was an original peer-reviewed research publication; and (d) the language in the article was English. We excluded abstracts from the review if the answer to any of these four criteria was no. If the answers to these questions were ‘yes’ and/or ‘cannot be determined’, then we included the article for full text review. Two authors (S.H. and T.P.) independently screened all titles and abstracts, and a third author (V.C.S.) randomly screened 25% of the abstracts. The authors used a verbal consensus process to resolve any disagreements and inconsistencies. 

The current review investigated the use of RS-based SCS as an objective measure to assess FVC in 2- to 10-year-old children. We excluded children below 2 years of age, as children’s dietary behaviors in infancy are different from toddlerhood and later; children transition from a milk-based diet to consuming solid foods and become more independent to feed themselves after their first year. Moreover, only 25% of U.S. infants consume any fruits and vegetables compared to 75% of toddlers [44]. We excluded 11- to 19-year-olds, as early adolescence starts approximately after 10 years and development continues until 21 years of age [45], which introduces changes in sex hormone status that can be a moderating factor for SCS status [46]. Moreover, children less than 10 years of age are less likely to reliably report their dietary intake (when compared to those over 10 years of age) and, thus, their dietary assessment is based on caregiver report versus self-report [9,10,47]. Therefore, upon recognizing the importance of an objective measure of FVC in this age group, we included literature discussing the use of RS in children between the ages of 2 and 10 years.

**Figure 1:**
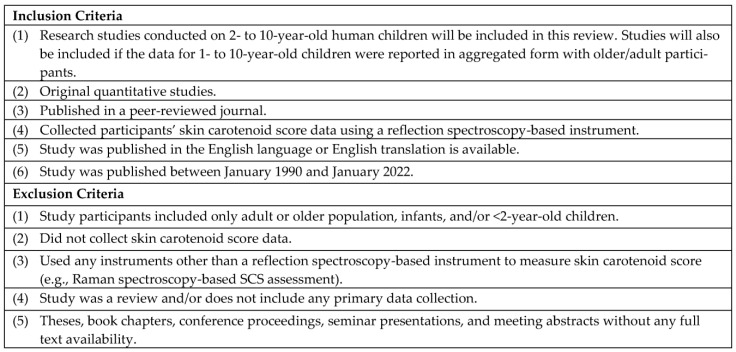
Inclusion and Exclusion Criteria for Selection of Studies.

During the full text review, the research team added one additional criterion to the four abstract screening inclusion criteria mentioned above: the study assessed the SCS using the pressure-mediated RS method. We included this additional inclusion criterion at the full text screening stage as the research article may not specify methods for SCS assessment in the title and abstracts due to conciseness. S.H. reviewed the articles for data extraction and qualitative synthesis. The author used DistillerSR for this stage as well. Figure 1 reports the detailed inclusion and exclusion criteria.

### 2.4. Data Extraction, Management, and Analysis

The research team extracted and synthesized data using another structured form with 18 items in DistillerSR (Appendix B), and did not exclude any articles in this stage. 

S.H. read the study objectives, methods, and results sections in the included articles to determine and summarize the current research uses of RS-based SCS assessment in children. S.H. also extracted data regarding the demographic characteristics of study participants (race, ethnicity, age, and biological sex), data collection settings, timepoints, device used for SCS assessment, methods for the SCS data collection, range of mean SCS for the participants, research study objectives pertaining to SCS, and statistical data reported to address individual study objectives. As ten out of the eleven articles used the VM^®^ to collect SCS data, the research team compared the reported methods for SCS data collection in light of the recently published standard guidelines recommended in Radtke et al. (2021) [48]. However, as these were not standard protocols based on scientific evidence and the recommendations were published in 2021, the research team did not consider adherence to these recommendations for risk of bias assessment of the included articles. 

For addressing the aims of the systematic review, we searched the objectives, methods, and analyses sections of the research articles to determine distributions of SCS across demographic groups, to identify potential non-dietary correlates for RS-based SCS, and to summarize the validity and reliability of RS-based SCS assessment in children as a proxy for FVC. 

In the current review, we investigated the criterion validity of RS-based SCS. Criterion validity is defined as an estimate of the extent to which a measure agrees with a standard [49]. Therefore, the criterion validity of RS-based SCS may include either validation with plasma carotenoids, which is the gold standard for carotenoid assessment [21], or fruit and vegetable intake measured with a standard dietary intake assessment tool. Additionally, the reliability of RS-based SCS includes inter-device reliability and repeatability of the SCS when using same RS device for a child.

#### Statistical Analysis

For the meta-analysis, we collected data regarding type and magnitude of statistical relationship between children’s SCS and FVC from the articles. We included four studies for meta-analysis: Liu et al. (2021) [50], Martinelli et al. (2021) [51], Nagao Sato et al. (2021) [52], and Takeuchi et al. (2022) [53]. The authors of the papers that reported the multivariate model were contacted to request the bivariate correlation statistics, confidence intervals, and *p*-values. After including the studies with unadjusted correlation between FVC and SCS, we excluded studies that did not report association between FVC and SCS. We used a random-effects model with the restricted maximum likelihood estimator for the between-study heterogeneity variance to pool the overall correlation between RS-assessed skin carotenoids and FVC using R (4.0.2) with the “robumeta” and “mmetafor” packages. We applied the Fisher Z-transformation to the correlation. We summarized the results in a forest plot, and the Higgin and Thompson heterogeneity index and Cochran Q test results are reported. To test for the presence of publication bias, funnel plots were visually inspected, and Egger tests and rank correlation tests were performed.

### 2.5. Risk and Bias Assessment

Two authors (S.H. and T.P.) independently assessed the quality of studies. Discrepancies were discussed and resolved through verbal consensus. For the observational, cohort, and cross-sectional studies, we assessed risk and bias using two tools: (1) the study quality assessment tool, U.S. National Heart Lung and Blood Institute for observational cohort and cross-sectional studies [54], and (2) the Strengthening the Reporting of Observational Studies in Epidemiology (STROBE) checklist [55]. For intervention studies, we used the NHLBI tool [54] to assess study quality. Based on the overall study quality, we categorized the studies into studies with low risk of bias, moderate risk of bias, and high risk of bias. 

## 3. Results

### 3.1. Overview of Search Results

**Figure 2:**
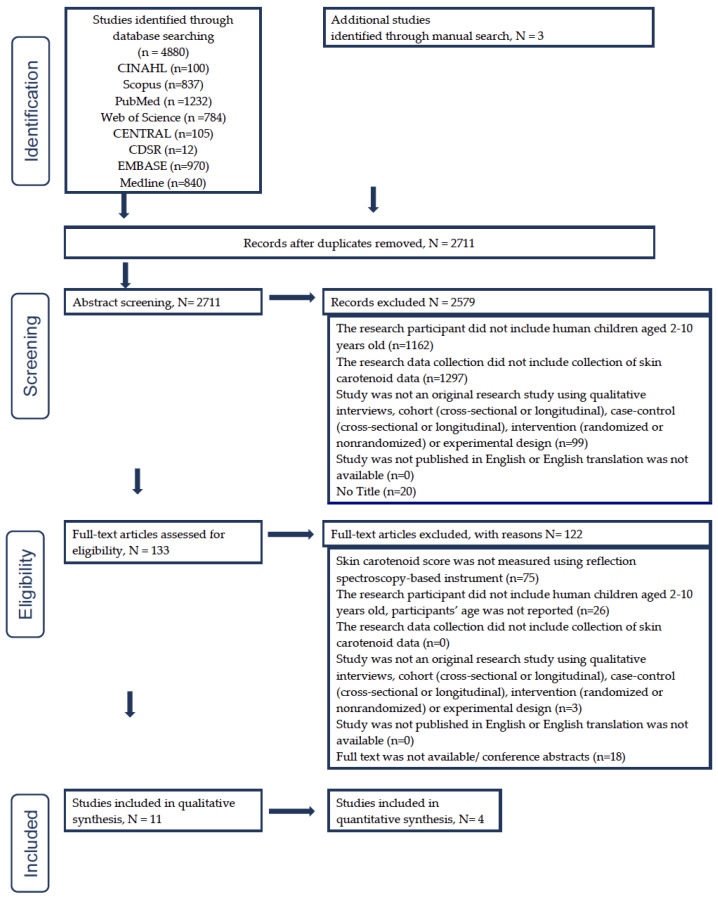
Study selection flowchart based on PRISMA (Preferred Reporting Items for Systematic Reviews and Meta-Analyses) statement guidelines [43].

The comprehensive literature search in eight search databases yielded 2711 unique articles, including three articles identified through the manual search. After the title and abstract screening, we excluded 2578 articles and had 133 articles for full text screening. Full text screening resulted in 11 articles eligible for the current review. The PRISMA figure (Figure 2) shows the step-by-step selection procedure and the numbers of articles we excluded under certain criteria.

### 3.2. Type of Studies

The 11 articles described nine unique research projects. Three out of the 11 articles were intervention studies—two were studies with a control group [56,57], one was an intervention study without a control group [58], and the remaining eight were cross-sectional studies [50,51,52,59,60,61,62]. The following sections discuss specific characteristics of each article (Table 1).

### 3.3. Study Characteristics

#### 3.3.1. Study Research Objectives

The research objectives for the eight cross-sectional studies varied and included identifying group (for age, ethnicity, biological sex, weight status) differences in children’s SCS [51,60,61]; refining procedures for collecting SCS data with RS in school settings [58]; and exploring associations between SCS and breastfeeding [50], diet [50,51,52,53,59,61], perceived stress [59], nutritional knowledge intervention [58], and seasonal variation [60]. The intervention studies (n = 3) provided nutrition education either to the parents of young children [57] or to the children [58,60], aiming to improve children’s FVC [56,57,58].

#### 3.3.2. Demographic Characteristics of Study Participants

Overall, all articles included in this review presented RS-based SCS results for 2–10-year-old children based on our inclusion criteria. Three articles, May et al. (2020) [61], Burkholder et al. (2021) [60], and Bayles et al. (2021) [56], had predominantly Black participants (77–86%); Nagao-Sato et al. (2021) [52] had 100% Latino participants; Bakırcı et al. (2019) [57] and Liu et al. (2021) [50] had a higher representation of White participants (68–80%) compared to the other studies. Additionally, Takeuchi et al. (2020) [53] had the highest representation for Asian participants (100%). Regarding participants’ biological sex, the studies included 48–57% male children [50,51,52,56,57,58,59,60,61].

#### 3.3.3. Time and Location

One article was published in 2014 [59] and all other articles were published from 2018 to 2022. One research project was set up in Korea and Germany [59], one research project was set up in Japan [53], and the other seven projects described in the remainder of the nine articles were conducted in the U.S. (Table 1) [50,51,52,56,57,58,60,61,62].

#### 3.3.4. Settings 

Collecting child-level SCS data took place in classrooms [56,58,60], laboratories [50], places of worship, and non-profit community centers [52].

#### 3.3.5. Data Collection Timepoints

Six cross-sectional studies reported one time point for data collection. Another two cross-sectional studies and three intervention studies reported three time points for data collection. Studies with three data collection time points had an average minimum span of 15.9 days [50] to a maximum of four months [58] between two data collection points. In these articles, the time of year (months and season) for SCS data collection was distributed across the year and was higher during the fall semester (September–December) compared to other months of the year. Burkholder et al. (2021) [60], Bayles et al. (2021) [56], Nagao-Sato et al. (2021) [52], and Jones et al. (2021) [58] reported data collection across the spring and fall seasons. 

#### 3.3.6. Methods Used for SCS Data Collection

The reported methods to collect SCS data from children were not similar across the studies. We found that none of the studies followed all recommendations for data collection. Radtke et al. (2021) [48] have published comprehensive recommendations to consider when assessing RS-based SCS from children. While most of the articles we reviewed did not include all these recommendations, the following recommendations were most frequently addressed: recording individual characteristics (age, sex, BMI, supplement use, chronic diseases) (n = 8), participants’ hand washing with soap and warm water or using hand wipes (n = 3), and calibrating the instrument every 1 h of operation or some calibration (n = 3). None of the studies reported using the following recommendations: acclimation period provided for the instrument in the new environment, any record taken for environmental conditions, and using the non-dominant ring finger for measuring SCS with RS (Table 2). 

#### 3.3.7. Devices Used 

All studies used the VM^®^ [38] to collect SCS, except Jung et al. (2014) who used a different pressure-mediated RS-based tool (Opsolution GmbH, Kassel, Germany) [63] (Table 3).

#### 3.3.8. Range of Mean SCS

For the studies using the VM^®^, the lowest mean SCS reported was 156.2 (standard deviation (±SD) = 78) [64], and the highest mean SCS was 380 (±SD = NR) [50] (Table 3).

### 3.4. Summary of Research Findings That Used RS-Based SCS Assessment in Children

Research objectives in the reviewed articles using the RS-based SCS in children fell into three categories: (1) identifying distributions of SCS across demographic groups, (2) identifying potential non-dietary correlates for RS-based SCS, and (3) summarizing the validity and reliability of RS-based SCS assessment in children as a proxy for FVC. The detailed results from the data extraction are provided below. 

#### 3.4.1. Distributions of SCS across Demographic Groups

Table 3 and Table 4 show the mean (±SD) SCS reported in the studies for different ages, biological sexes, and ethnic and racial groups of children and the relationship between demographic characteristics and SCS, respectively. Seven out of the eight cross-sectional studies [50,51,52,59,60,61,62] (n = 8) reported children’s SCS distribution across various demographic variables (e.g., age, biological sex, race and ethnicity, income, and mother’s education).

(1) Biological Sex. Mean (±SD) SCS reported in two studies conducted on the same sample were 282.5 (±75.1) for male and 243.4 (±88.9) for female children [60,61]. Studies (n = 5) investigating the influence of biological sex on SCS found higher SCS in male participants compared to females in three studies [52,60,61] and no significant difference in one study [51]. Jung et al. (2014) [59] reported that the relationship between biological sex and SCS may be moderated by race/ethnicity. That is, they found significantly higher SCS for German male participants compared to German female participants, but significantly lower SCS for Korean male participants compared to Korean female participants [59].

(2) Age. Only Burkholder et al. (2021) reported SCS for 3-, 4-, and 5-year-old children, where 3-year-old children had the lowest SCS (mean ± SD = 241 ± 79.4) and 5-year-old children had the highest SCS (mean ± SD = 339 ± 137.5) [60]. Two studies reported higher SCS in older age groups compared to younger groups [59,60], and two others reported no significant differences in SCS between age groups [51,52].

(3) Race, Ethnicity, and Nationality. Regarding racial and ethnic group differences, May et al. (2020) reported White children (281 ± 91.6) had comparatively higher mean SCS than children from Latino (225 ± 95), Black (265.2 ± 84.4), and other racial groups [61]. Two studies found no significant differences in SCS across racial and ethnic groups [51,61]. Ermakov et al. (2018) reported some differences but did not report the type and magnitude of the relationship between SCS and race/ethnicity [62]. Jung et al. (2014) found higher SCS in Korean participants compared to German participants; however, the SCS scale used in this study was different from other studies included in this review, thus precluding direct comparison.

(4) Income, Employment Status, and Mother’s Education. Among studies [50,51,52,62] (n = 4) exploring distribution of SCS across different income and employment status, one study reported children from schools categorized as high income level (201 ± 80) had significantly lower SCS than children from schools with a low income level (221 ± 59) [51]. Ermakov et al. (2018) reported some differences in SCS across different income levels; however, details about type and magnitude were not reported [62]. Two other studies found no significant differences in SCS for income and employment status [50,52]. Liu et al. (2021) reported children’s SCS was not significantly different for mothers’ level of education [50]. 

#### 3.4.2. Potential Non-Dietary Correlates of Children’s RS-Based SCS

Seven out of 11 articles explored associations between non-demographic individual and environmental-level factors with RS-based SCS. Table 4 lists these factors and their relationships with SCS reported in the studies. 

(1) Perceived Stress. Jung et al. (2014) found Korean children with higher perceived stress had lower SCS than children with lower perceived stress; however, no confidence intervals or *p*-values were reported for this relationship [59].

(2) Seasonality. Two cross-sectional studies [52,60] and one intervention study [56] reported that SCS was significantly lower during the winter season [56,60] compared to summer [60] and during the fall season [52,56,60], The change in SCS between summer and fall was not significant [60]. Contrary to these three studies, Jones et al. (2021) reported higher scores in spring compared to fall in response to a school-based nutrition intervention [58].

(3) Overweight/obesity. Mean SCS reported for children with overweight/obese weight status (as defined by body mass index) ranged from 218 (±98) [52] to 274.6 (±75) [61] and for children with a healthy weight was from 235 (±90) [52] to 260.4 (±89.1) [61]. Studies (n = 4) connecting weight status with lower SCS have reported mixed findings. Three studies found non-significant group differences for SCS between healthy/underweight and obese/overweight groups [52,58,61]. Liu et al. (2021) reported a significant inverse association between BMI percentile and SCS [50]. Jung et al. (2014) also reported that SCS was lower with higher body weight [59]. Additionally, birth weight for gestational age percentile did not have any significant correlation with SCS [50].

(4) Body Fat Percentage. Higher body fat percentage and higher visceral adipose tissue had inverse correlations with SCS regardless of BMI percentile in the single study including body composition [50]. 

(5) Breastfeeding Exposure. Total breastfeeding duration, exclusive breastfeeding duration, and nonexclusive breastfeeding duration were not significantly associated with SCS [50]. 

(6) Nutritional Knowledge. Changes in school-aged children’s nutritional knowledge were not associated with their SCS [58].

#### 3.4.3. Summary of the Validity and Reliability of RS-Based SCS Assessment in Children as a Proxy for FVC

Six out of 11 articles examined associations between FVC and SCS. These articles reported that carotenoid intake [50,52,53], FVC [50,51,52,53,59], and vegetable consumption [51,53] were positively associated with SCS. Furthermore, liking of fruit and vegetables [61], total energy intake [50], and dietary intake of food groups other than fruit and vegetables [53] had no significant association with SCS [52]. Additionally, 5 out of 11 articles reported an association between FVC measured with other assessments and RS-based SCS (Table 4) [50,51,52,61]. Specifically, articles reported the following assessments for measuring FVC: self-reported questionnaire [53,59], School Physical Activity and Nutrition (SPAN) survey [51], pictorial fruit and vegetable liking tool [61], 24 h dietary recall interviews, and parent-reported seven-day diet record [50]. FVC measured via 24 h dietary recall [52], the SPAN survey [51], and total dietary carotenoids measured via seven-day diet record [50] and 24 h dietary recall [52] were found to be positively associated with SCS. The correlation value (r) ranged from 0.17 to 0.25 (*p* < 0.05) (Table 2). Liking of fruits and vegetables measured using the pictorial liking tool was not associated with SCS [61]. None of the reviewed studies used food frequency questionnaires to examine associations with SCS. 

None of the studies reported reliability of RS to measure SCS in children. Nagao Sato et al. (2021) [52] reported that researchers repeated the SCS measure procedure when the average score measured was below 200, with the reasoning that intra-device reliability of the VM^®^ device was originally established when the lowest score was 200 in Ermakov et al. (2018) [62]. 

### 3.5. Meta-Analysis

**Figure 3:**
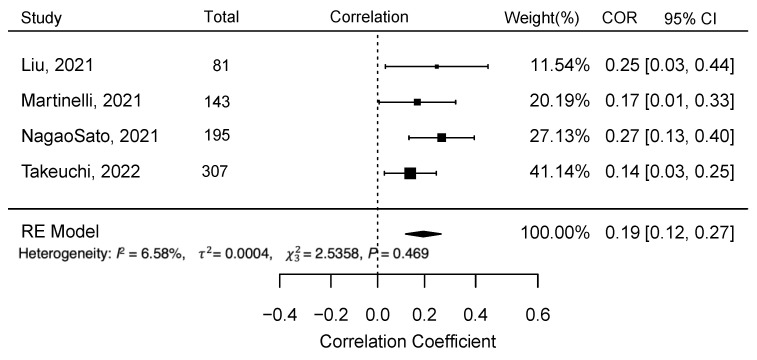
Meta-analysis results for the four studies– Liu et al. (2021) [50], Martinelli et al. (2021) [51], Nagao Sato et al. (2021) [52], and Takeuchi et al. (2022) [53], to examine the correlation between reflection spectroscopy-based skin carotenoid score with fruit and vegetable consumption in children (*Abbreviation:* COR = Correlation, CI = Confidence Interval).

**Figure 4:**
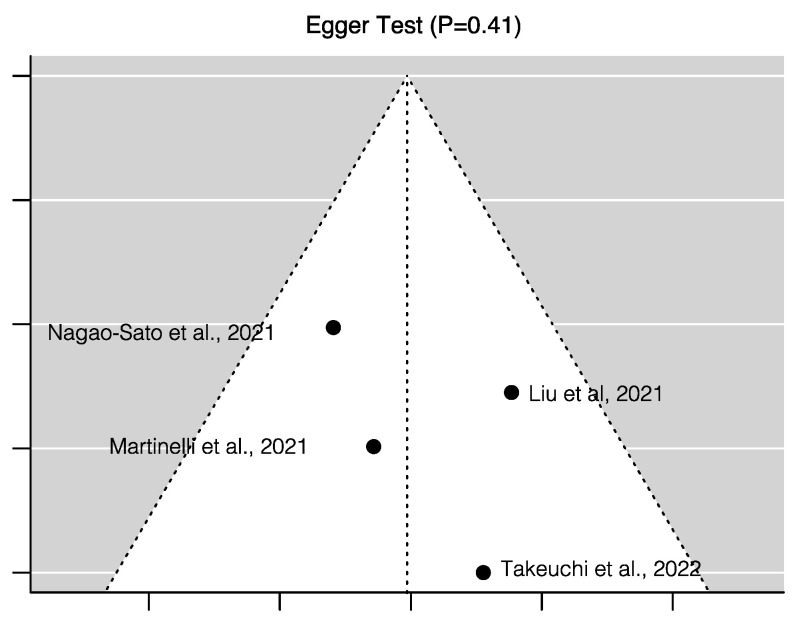
Risk of publication bias assessment for the four studies included in the meta-analysis– Liu et al. (2021) [50], Martinelli et al. (2021) [51], Nagao Sato et al. (2021) [52], and Takeuchi et al. (2022) [53], to examine the correlation between reflection spectroscopy-assessed skin carotenoid score with fruit and vegetable consumption in children.

Figure 3 shows the random-effects model for all four studies considered for the meta-analysis. The random-effects model yielded an overall Pearson correlation of *r* = 0.20 (95% CI 0.12 – 0.27); *I*^2^ = 6.58 (95% CI 0 – 89.84; *p* = <0.0001). The overall *z*-test of the pooled correlation was statistically significant (*p* < 0.0001). The *Q*-statistic for between-study variance was non-significant (*p* = 0.47), hence the null hypothesis for whether the studies share a common effect size could not be rejected. There was no evidence for publication bias of studies. Neither the rank correlation (*p* = 0.75) nor Egger’s regression test (*p* = 0.41) was statistically significant, which is consistent with the assumptions from the funnel plot (Figure 4). 

### 3.6. Risk and Bias Assessment

Risk and bias assessment using the NHLBI tool [54] for the three intervention studies indicated that two had moderate risk of bias and another had high risk of bias. Not including a control group, not randomizing the intervention and control group, convenience sampling, small sample size, high attrition (22–30%), high differential drop-out rate between two groups (20%), and only considering participants who completed the full intervention were contributing factors towards higher risk of biases. Conversely, consistent implementation of interventions across studies, providing justifications for sample size, analyzing and reporting baseline group differences between intervention and control groups, analyzing outcomes for subgroups, and using appropriate statistical analyses were contributing factors towards strengths of methodology. 

Risk and bias assessments for cross-sectional studies using the NHLBI tool dictated that two studies had high risk, one study had moderate risk, and four studies had low risk of bias. Studies categorized as having high risk of bias had the following weaknesses: lack of description on self-reporting or parent proxy for child participants’ diet history, lack of reports about recruitment, one data collection point, and no consideration of confounding variables in the analyses. The use of retrospective measurement for exposure variables contributed to one cross-sectional study having moderate risk of bias. Studies categorized as having low risk of bias reported appropriate statistical analyses, considered confounders when applicable, reported recruitment criteria, and provided detailed justification of exposures and outcome measures. 

Using the STROBE checklist [55], the following weaknesses were observed for the eight cross-sectional studies: not mentioning the study design early in the article, inconsistency in reporting the number of participants at each stage of the study starting from recruitment until completing data collection, not addressing missing data in the analyses, not reporting sample size justification, not clearly reporting results with references to objectives, and lack of discussion regarding generalizability. Additionally, none of the studies reported sensitivity analysis. 

## 4. Discussion

The current review focused on the use of RS to measure SCS in 2- to 10-year-old children. Researchers are increasingly using RS to assess children’s SCS in schools [51], child care [56,60,62], and other community settings, such as at places of worship [52] and at public libraries [57] due to the convenience and non-invasiveness of the tool. The current systematic review provides several implications for future research, policy, and practice for using RS-based SCS as a dietary assessment tool for children. 

### 4.1. Validity and Reliability 

The emerging research shows promising evidence regarding the validity of RS-based SCS for estimating FVC in children. Previous criterion validation studies found that RS-based SCS is strongly correlated with plasma carotenoids (*r* = 0.70) in adult participants [32]. The current review did not find any criterion validation studies with plasma carotenoids in children. However, our meta-analyses results showed criterion validity of RS-based SCS with small but significant correlations with children’s FVC (*r* = 0.2, *p* < 0.0001). This value is comparable with the studies conducted in adults (*r_adults_* = 0.27) [32]. Therefore, the current review findings suggest future research is needed to determine criterion validity of RS-based SCS with plasma carotenoids in children.

The current review also suggests the importance of validating RS-based SCS in children with racial and ethnic diversity [52,56,60,61]. This suggestion is reflected by the findings of Takeuchi et al. (2022), as they reported a higher range of SCS (138–822) [53] for Japanese children in their study compared to the other studies conducted in the U.S. children [51,56,60]. The higher range of SCS in children living in different countries can be linked to the differences in biological biomarkers due to genetic differences [65,66], ethnicity, or overall diet quality [67], which is an important area to explore for future validation studies. 

The reliability of RS devices has not been investigated in children, but both intra- and inter-device reliability [68] have been established in adults in lab [62] and community settings [69]. Additionally, the VM^®^ offers two modes of operations—single- and three-scan mode [38]. The three-scan mode has been suggested to improve the accuracy and reliability of the VM^®^ reading up to two-fold over the single-scan method [62]. However, of the 11 studies included in this review, only two used the three-scan method [60,64], likely due to the challenges of having young children insert and reinsert their fingers into the device three times. More research is likely needed to explore the potential level of variability, compliance, withdrawals, and accuracy produced with each of these methods for children.

### 4.2. Implications for Future Research 

The first step for using RS-based SCS as a fruit and vegetable screener in children is to establish how RS-based SCS can be translated to the amount of daily FVC in children. Such information is currently available for RRS but not RS. For example, for RRS, one study demonstrates that 30,489.8 (±4667.1) RRS units of SCS in children translates into 0.5 cups of FV/day consumption [35]. Therefore, future randomized control trials are recommended to establish reference points for using RS-based SCS to estimate children’s daily FVC, similar to RRS. Additionally, a randomized dietary study (e.g., high-carotenoid vs. low-carotenoid diet) would improve understanding of the sensitivity of RS in children [22].

The current review also emphasizes the need for future studies using RS-based SCS in children to use the standard protocol recommended by Radtke et al. (2021) [48]. Following a standardized protocol will allow researchers to contribute to a shared population-level database to establish reference points and compare findings from different studies. Additionally, future research studies may consider reporting about nutrient supplement usage, as previous intervention studies reported a significant and consistent increase in RS-based SCS in adults after taking high doses of lutein and zeaxanthin supplements [70]. 

In this review, the most common non-dietary correlates for children’s RS-based SCS (n ≥ 5 articles) were children’s weight status and biological sex. Studies reported an inverse correlation between SCS and indicators of body weight status, specifically body fat percentage in children [50,59], similar to research conducted in adults [71,72]. However, the relationship between BMI percentile and children’s SCS was inconsistent, mainly because children’s BMI percentile is a poor indicator of body fat percentage [73]. The current review also cited significant higher SCS in males than female children in the U.S. [60,61] and Germany [59]. However, SCS was significantly lower in males compared to females in Korean participants [59], and no significant relationship was reported between sex and SCS in early adolescents (7–12-year-olds) in the U.S. [52] and Japan [53]. Inconsistencies in the relationship between RS-based SCS and biological sex have been linked with ethnic differences in carotenoid biodistribution in males versus females and sex hormone status [46]; however, these inconsistencies warrant future research.

Future research studies should also consider children’s age, type of setting, and seasonal variation as covariates for tracking changes in RS-based SCS over multiple data collection timepoints. Though children’s age was not a significant covariate for SCS across the reviewed studies, age may be a moderator for the relationship between seasonal variation and SCS. Preschool children’s diet quality is more dependent on their caregivers compared to older children, and they also consume more fruits and vegetables in the childcare setting than at home and outside [74]. Therefore, preschool children may face low availability of carotenoid-rich FV during the winter season (winter break) compared to summer and fall [56,57], leading to a decrease in their SCS. However, older children with nutrition education may have more control over their consumption patterns than preschool children, leading to an increase in their SCS after winter [58]. 

This review suggests refining research questions and study designs when utilizing RS-based SCS as a proxy measure for FVC in children. The designs for 82% of the reviewed studies were cross-sectional, and future studies should consider including more robust study designs. Current evidence suggests that researchers are likely to make more accurate conclusions when using RS as a within-person screening tool to assess changes in diet quality over time versus comparing between groups, due to inter-individual variation in carotenoid biodistribution and covariates. 

Finally, the current evidence also underscores RS-based SCS as a promising assessment tool to evaluate policies and interventions to improve the dietary intake of fruit and vegetables for the prevention of childhood obesity and chronic diseases, such as cardiovascular disease, diabetes, and cancer. Therefore, RS-based SCS has the potential to be utilized as a non-invasive biomarker [75,76,77] and population health outcome measure for obesity, cancer, and chronic disease prevention in young children and adults.

### 4.3. Implications for Policy and Practice 

Given that using RS-based SCS is convenient, and SCS is an established biomarker for FVC [20,23], it offers a promising avenue for evaluating the impact of federal policies and nutrition assistance programs targeting improving children’s dietary behaviors [58]. This review highlighted the efficiency of RS-based SCS to track changes in children’s SCS [56,58,60]. Therefore, RS-based SCS could inform program evaluation in educational institutions serving children from low-income families and minority groups, for programs such as the Supplemental Nutrition Assistance Program Education (SNAP-Ed), childcare settings participating in the Child and Adult Care Food Program (CACFP), and Head Start. Moreover, federal food and nutrition assistance programs involving regular check-ins with their participants, e.g., the Special Supplemental Nutrition Program for Women, Infants, and Children (WIC) and SNAP-Ed, may use the RS-based SCS as a child-level effectiveness outcome measure to track improvement in child dietary intake along with household food availability. 

### 4.4. Limitations 

The current review’s methodology has the following limitations. First, the scope of our review was limited to 2- to 10-year-old children. However, because some papers included 2–10-year-old children along with adults but did not provide results separated by age, we included the results with a mixed-age group of participants ranging from children to older adults. Next, though several studies were published before 2021, we compared the reported data collection protocol with more recently published standard guidelines for RS-based SCS, which have not yet been established as evidence-based practices. In addition, study inclusion was limited to those studies published in the English language. Thus, international studies may have been missed. For the meta-analysis, because of the low number of studies (n = 4), the *I*^2^ value is subject to bias [78] and should be interpreted with caution.

## 5. Conclusions

RS-based SCS is a valid method to quantify skin carotenoids for children’s FVC estimation, with the potential for evaluating nutrition policies and interventions. However, future research should establish how RS-based SCS can translate to the amount of daily FVC in children. Further, studies using RS-based SCS should follow a standardized protocol and consider correlates for improved validation and establishing reference points. Future research should determine inter-device reliability and establish criterion validity of RS-based SCS with plasma carotenoids in children. 

## Figures and Tables

**Table 1:** Demographic characteristics of study participants and data collection.

Study/Location	Objectives	Participant Characteristics (Total, % Male, % Race/Ethnicity)	Children’s Age (In Years) Range (M ± SD)	Weight Status	Setting for Data Collection	How Many Times the Skin Carotenoid Data Were Collected; Average Time between the Data Collection Points	Data Collection Months
Controlled Intervention Study							
Bakırcı et al. (2019); TX, USA [57]	To determine post-nutrition intervention increase in skin carotenoid score	n = 30, 50% male; Intervention (I): White 80%, Hispanic/Latino 7%, Asian/Pacific Islander 7%, Other 7%; Control (C):White 67%, Hispanic/Latino 20%, Black 7%, Asian 7%	3–5; (I = 3.6 ± 1.4, C = 3.8 ± 0.8)	I: Healthy weight 100%; C: Healthy weight 80%, Overweight/obese 20%	Library	3 times; 5 weeks	NR *
Bayles et al. (2021); NC, USA [56]	To investigate effectiveness of vegetable exposure through food-based science, technology, engineering, arts, mathematics (STEAM) learning activities in classroom to increase liking for vegetables and objectively assessed FV intake.	N = 112, 49% male; Intervention (I): Black 85.7%, White 4.1%, Hispanic/Latino 2%, Asian 0%, Other 8.2%; Control (C): Black 76.6%, White 6.3%, Hispanic/Latino 9.4%, Asian 1.6%, Other 4.7%.	3–5; (I = 3.8 ± 0.6, C = 3.6 ± 0.6)	I: BMI-Z (0.7 ± 1.4); Underweight 4.1%, Normal 57.1%, Overweight 10.2%, Obese 28.6%; C: BMI-Z (0.7 ± 1.4); Underweight 4.7%, Normal 50%, Overweight 20.3%, Obese 25%.	Head Start	3 times; 2 months	September 2019–February 2020
Intervention Study without Control Group							
Jones et al. (2021); CA, USA [58]	(a) To determine efficiency of the VM^®^ to assess changes in FV consumption in a school-based intervention (b) To refine the protocol for using the VM^®^ in low-income schools to collect SCS	n = 35, 48.6% male; American Indian/Alaskan Native 2.9% Asian/Pacific Islander 20% White 34.3% Mixed 14.3% Other 2.9% Latino/Hispanic 22.9%	9–10	BMI (64 ± 30.1)	Supplemental Nutrition Assistance Program Education qualifying schools	3 times; 4 months	Fall 2018, spring 2019, fall 2019
Cross-sectional Study							
Burkholder et al. (2021); NC, USA [60]	To investigate the change in skin carotenoid score regarding seasonal variation, availability of fruit and vegetables, and across different ages and genders	n = 112, 57% male; African American 80.5%	3–5 (NR)	BMI-Z (0.7 ± 1.4); Overweight 16%, Obese 27%	Head Start	3 times; 2 months	October 2018–February 2019
Ermakov et al. (2018); CA, USA [62]	To understand the distribution of reflection spectroscopy-based skin carotenoid score in preschool children	n = 947, NR	2–5 (NR)	NR	Childcare Center	1 time; NA *	Fall 2017–spring 2018
Jung et al. (2014); Korea and Germany [59]	To investigate how the nutritional and cultural habits including stress behavior are reflected in the antioxidant status of the skin	n = 714, 53.2% male; Korean 53.5%, German 46.5%	Total 7–75; Percent of children in the total sample (n = 11.9%) 7–13	Underweight 3.5%, Overweight 19.6%, Obese 2.9%	NR	NR; NA	June–August, NR
Liu et al. (2021); IL, USA [50]	To investigate interrelations between breastfeeding exposure, weight status, adiposity, and carotenoid status among children at school age	n = 81, 51.9% male; Hispanic 6.2%; Asian 14.8%, Black or African American 6.2%, White 67.9%, Mixed/other 11.1%	7–12 (9.4 ± 1.6)	Normal weight 73%, Overweight or obese 22%	Laboratory	3 times, 15.9 days	NR
Martinelli et al. (2021); AZ, USA [51]	(a) To assess feasibility of using Veggie Meter^®^ in an elementary school setting; (b) the distribution of skin carotenoids among elementary-school-age children; (c) exploring variation in skin carotenoid score with demographic characteristics; (d) To compare skin carotenoid score with self-reported FV intake on the previous day	n = 143, 47.6% male; Hispanic 45.5%, White 37.1%, Other 17.5%	9–11	NR	School	1 time, NA	November 2019
May et al. (2020); NC, USA [61]	(a) Association between skin carotenoid score and fruit and vegetable intake. (b) Age-, gender-, and weight category-related group differences in skin carotenoid score	n = 112, 57% male; Black 81.3%, White 5.4%, Other 13.3%	3–5 (4.1 ± 0.5)	BMI (67.5 ± 32.1) Overweight or obese 43%, Healthy weight 57%	Head Start	1 time, NA	October–December 2018
Nagao-Sato et al. (2021); MN-WI, USA [52]	To explore association between reflection spectroscopy-based skin carotenoid score with fruit and vegetable intake controlling for potential confounding factors	n = 195, 50% male; Latino 100%	10–14 (12 ± 1.4)	Underweight/Normal weight 43%, Overweight/obesity 57%	Three churches and two Latino-serving nonprofit community centers	1 time, NA	January, February, March, September, and October; 2017–2020.
Takeuchi et al. (2022), Japan [53]	To evaluate the association between skin carotenoid score with fruit and vegetable intake and other dietary habits in children	n = 328, 50.8% male; Japanese	10 (NR)	CD	NR	1 time, NA	January, 2020

* NR = not reported; BMI = body mass index; BMI-Z = BMI for age z-score; NA = not applicable.

**Table 2:** Reported Veggie Meter^®^ protocol to assess skin carotenoids in included studies compared to recommended protocol [41].

Recommendations for Using Veggie Meter^® a^	Studies Following Recommendation
1. Mentioned acclimation period for the instrument in the new environment	None
2. Calibrating the instrument every 1 h of operation or some calibration	Nagao-Sato et al. (2021) [52]; Martinelli et al. (2021) [51]; Liu et al. (2021) [50]; Jones et al. (2021) [58]
3. Measuring skin carotenoid scores using triplicate (three-scan) method	Bakırcı et al. (2019) [57] Martinelli et al. (2021) [51]
4. Recording individual characteristics: age, sex, BMI, supplement use, chronic diseases, etc.	Bayles et al. (2021) [56]; Burkholder et al. (2021) [60]; Ermakov et al. (2018) [62]; Bakırcı-Taylor et al. (2019) [57]; Liu et al. (2021) [50]; Martinelli et al. (2021) [51]; May et al. (2020) [61]; Nagao-Sato et al. (2021) [52]; Jones et al. (2021) [58]
5. Recording environmental conditions	None
6. Mentioned the use of nondominant ring finger	Jones et al. (2021) [58]
7. Participants’ hand washing with soap and warm water or using hand wipes	Burkholder et al. (2021) [60]; Bayles et al. (2021) [56]; Ermakov et al. (2018) [62]; Jones et al. (2021) [58]
8. Reported using same instrument for a group of participant/tracking instrument for repeated measures of skin carotenoid score for same participant	Martinelli et al. (2021) [51]; Nagao-Sato et al. (2021) [52]

^a^ Radtke et al. (2021) [48] have reported standardized protocol to use the Veggie Meter^®^ in future research efforts. This set of recommendations was assembled based on the Veggie Meter^®^ operating procedures from the developers; therefore, findings from Jung et al. (2014) [59] were not included in this table.

**Table 3:** Validity of reflection spectroscopy-based skin carotenoid assessment in children as a proxy for fruit and vegetable consumption.

Study, Device Used	Skin Carotenoid Score (M ± SD); Range	Operationalization of Fruit and Vegetable Consumption	Assessment Used	Correlation
Bakırcı et al. (2019); Veggie Meter^®^ [57]	NR	Accessibility and availability of fruit and vegetables at home	(a) Parent-reported online survey: *Focus on Veggies*. This is a two-item questionnaire: Item 1—Child behaviors measured with six questions; Item 2—Parent behavior measured with four questions. (b) Electronic food photos: Parents were trained and reminded to send photos via text or email of each meal and snack the child ate on the selected days using their own mobile devices. The goal for this measure was to determine the effect of intervention on fruit and vegetable accessibility. The photos were manually coded to count total fruits and vegetables served in captured meals and snacks.	NR
Bayles et al. (2021); Veggie Meter^®^ [56]	I = 267.2 ± 100.2; Seasonal Variation: Fall (268.6 ± 13.2); Winter (271.3 ± 12.5); After Winter Vacation (267.8 ± 11.3). C = 265 ± 67.5; Seasonal Variation: Fall (270.9 ± 12.1); Winter (275.6 ± 11.5); After Winter Vacation (229.6 ± 10.3).	Fruit and vegetable liking score	Self-reported pictorial liking tool was used to identify preschool children’s liking for nine target vegetables (broccoli, cauliflower, spinach, radish, sweet potato, cucumber, tomato, carrot, and pea pod) and other foods commonly consumed by children (e.g., hotdog, yogurt).	NR
Burkholder et al. (2021); Veggie Meter^®^ [60]	Total 266 ± 82.9; NR Sex: Male 282.5 ± 75.1 Female 243.4 ± 88.9 Age: 3 years 241 ± 79.4; 4 years 267 ± 68.8; 5 years 339 ± 137.5. Seasonal Variation: Fall (267.6 ± 8.7); Before Winter Vacation (273.8 ± 9.3); After Winter Vacation (228.7 ± 10.3).	Fruits and vegetable availability across summer, fall, and winter	Head Start menu for study duration	NA
Ermakov et al. (2018); Veggie Meter^®^ [62]	Total 380 ± NR; NR	NA		NA
Jung et al. (2014); LED-based compact scanner system, Opsolution GmbH, Kassel, Germany [59]	Korean 5.81 ± 0.1; German 4.62 ± 0.1; Immigrant Korean 4.77 ± 0.2	Fruit and vegetable consumption	Self-reported questionnaire asking about sex, age, BMI, subjective stress level (personal and occupational), vegetable and fruit consumption, smoking, and Korean or Western dietary habits.	NR
Jones et al. (2021); Veggie Meter^®^ [58]	Seasonal Variation: Fall 2018 156.2 ± 78; Spring 2019 211 ± 76.5; Fall 2019 195.4 ± 64.1.	NA	NA	NA
Liu et al. (2021); Veggie Meter^®^ [50]	Total 304.1 ± 100.7; NR	Total carotenoid intake	Seven-day diet record, parent- and participant-reported	*r* = 0.25; *p* < 0.05
Martinelli et al. (2021); Veggie Meter^®^ [51]	Total 210 ± 72; 34–447 High-income school 201 ± 80 Low-income school 221 ± 59	Fruit and vegetable consumption	Self-reported School Physical Activity and Nutrition (SPAN) survey	*r* = 0.17, *p* = 0.042
May et al. (2020); Veggie Meter^®^ [61]	Total 266 ± 82.9; NR Sex: Male 282.5 ± 75.1 Female 243.4 ± 88.9 Race: Black 265.23 ± 84.4 White 281 ± 91.6 Other 263 ± 77.9 Weight Status: Healthy 260.4 ± 89.1 Overweight/Obese 274.6 ± 75	Fruit and vegetable liking score	Self-reported pictorial liking tool was used to identify preschool children’s liking for 2 fruits and 10 vegetables.	NS range of correlation for the liking score of 12 fruits and vegetables, *r* = [−0.1 to 0.1]; range of *p* values [0.3 to 1]
Nagao-Sato et al. (2021); Veggie Meter^®^ [52]	Total 225 ± 95; (All Latino) Sex: Male 229 ± 89 Female 221 ± 100 Annual Household Income: USD <25,000 231 ± 105 USD ≥25,000 218 ± 86 Weight Status: Underweight/normal weight 235 ± 90 Overweight/Obese 218 ± 98 Seasonal Variation: Fall 242 ± 102; Winter 211 ± 86;	Fruit and vegetable intake, total carotenoid intake	Three 24 h dietary recall interviews were completed, where one interview was conducted in-person and two others were via phone calls over three weeks.	Fruit and vegetable intake, *r_f_* = 0.27, *p* < 0.05 Total carotenoid intake, *r*_2_ = 0.25, *p* < 0.05.
Takeuchi et al. (2022); Veggie Meter^®^ [53]	Total 349 ± 104; 138–822	Fruit intake, green-yellow vegetable intake, light yellow vegetable intake	Child-reported food frequency questionnaire administered by guardians	Fruit intake, ß_1_ (unstandardized beta coefficient) = 13.7, *p* = 0.04 Green-yellow vegetable intake, ß_2_ (Unstandardized beta coefficient) = 16.0, *p* = 0.01 Light yellow vegetable intake, ß_3_ = −5.17, *p* = 0.56

**Table 4:** Potential non-dietary correlates and their relationship with children’s RS-based skin carotenoid score.

Covariates	Types of Relationship Found in Studies ^a^		
	Positive	Inverse	None
Demographic Characteristics			
Sex (Female = 0, Male = 1)	Burkholder et al. (2021) [60] Jung et al. (2014) [59] May et al. (2020) [61]		Martinelli et al. (2021) [51] Nagao-Sato et al. (2021) [52] Liu et al. (2021) [50] Takeuchi et al. (2022) [53]
Age	Burkholder et al. (2021) [60] Jung et al. (2014) [59]		Martinelli et al. (2021) [51] Nagao-Sato et al. (2021) [52]
Race (Other = 0, White = 1)			Martinelli et al. (2021) [51] May et al. (2020) [61]
Ethnicity (Non-Hispanic = 0, Hispanic = 1)			Martinelli et al. (2021) [51]
Nationality (German = 0, Korean = 1)	Jung et al. (2014) [59]		
Income	Martinelli et al. (2021) [51]		Nagao-Sato et al. (2021) [52] Liu et al. (2021) [50]
Employment status			Nagao-Sato et al. (2021) [52]
Mother’s education			Liu et al. (2021) [50]
Body Weight			
Overweight/obesity/BMI percentile		Jung et al. (2014) [59] Liu et al. (2021) [50]	May et al. (2020) [61] Nagao-Sato et al. (2021) [52] Jones et al. (2021) [58]
Percent body fat		Liu et al. (2021) [50]	
Visceral adiposity		Liu et al. (2021) [50]	
Weight for gestational age percentile			Liu et al. (2021) [50]
Others			
Seasonal variation (Winter)			Nagao-Sato et al. (2021) Bayles et al. (2021) [56] Burkholder et al. (2021)
Home food availability and accessibility			Nagao-Sato et al. (2021) [52]
Breastfeeding exposure			Liu et al. (2021) [50]
Nutritional knowledge			Jones et al. (2021) [58]
Exercises	Takeuchi et al. (2022) [53]		
Passive smoking			Takeuchi et al. (2022) [53]

^a^ Ermakov et al. (2018) [62] have reported significant differences in SCS for race/ethnicity, income, and seasonal variation but do not mention direction, hence this was not reported in Table 4.

## Data Availability

No new data were created or analyzed in this study. Data sharing is not applicable to this article.

## Appendix A. Search Strategy

Search Sets Forwarded for Review: #1|#23 Database: Ovid MEDLINE(R) and Epub Ahead of Print, In-Process, In-Data-Review & Other Non-Indexed Citations and Daily <1946 to 6 May 2021> Search Strategy:
--------------------------------------------------------------------------------
1 veggie$ met$.mp. (8)
2 nuskin$.mp. (1)
3 exp Spectrum Analysis/(559417)
4 (spectroscop$ or spectometr$ or spectrum$ analy$ or spectophotometr$).mp. (606114)
5 (reflectance$ or biophotonic$ or bio-photonic$).mp. (25352)
6 (optical$ adj2 (detect$ or assess$ or sens$)).mp. (14533)
7 (raman$ adj2 (microscop$ or imag$ or resonan$)).mp. (10190)
8 or/2–7 (837113)
9 exp Skin/(229427)
10 (skin$ or derm$ or epiderm$).mp. (1153364)
11 exp Blood/(1124991)
12 (blood$ or plasma$ or serum$ or sera).mp. (4841063)
13 or/9–12 (6269201)
14 8 and 13 (118002)
15 exp Carotenoids/(87199)
16 caroten$.mp. (40527)
17 (alphacaroten$ or betacaroten$).mp. (79)
18 (astacen$ or cryptoxanthin$ or betacryptoxanthin$ or canthaxanthin$ or fucoxanthin$ or lutein$ or lycopen$ or zeaxanthin$).mp. (75804)
19 or/15–18 (166545)
20 14 and 19 (1024)
21 ..l/20 lg = en (978)
22 ..l/21 yr = 1990-current (760)
23 remove duplicates from 22 (760)
***************************

## Appendix B. Full Text Data Extraction Form

1. Country in which the study took place ………….
2. Research Question/Objective/Hypothesis ………….
3. Research method/study design (Select from the following options)
a. Cross-sectional study
b. Cohort
c. Case-control study
d. Intervention study without control group
e. Intervention study with control group
4. Total sample size ……….
5. Participant Characteristics
a. Gender …………
b. Race/ethnicity ……………
c. Socioeconomic Status …………
d. Weight status …………
e. Age (mean, SD, range) …………….
6. Data collection setting (Select from the following options)
a. Child care/day care settings
b. Head Start
c. Preschool
d. School
e. Laboratory
f. Other, please specify ……….
7. Duration of data collection over the year, mention particular month or season ………
8. Over how many months or years data were collected?............
9. Dependent variable ………………
10. Assessment/measure/tools used for measuring dependent variable ………….
11. Independent variable ……………
12. Assessment/measure/tools used for measuring independent variable ………….
13. Statistical analysis used (e.g., Psychometric analysis of RS-based skin carotenoid in children as a proxy for fruit/vegetables consumption) ……………
14. Reported correlates, covariates, and confounding factors ……………
15. Test statistic values …………
16. Research Implications (areas and opportunities for research with RS-based carotenoid assessment in children ………….
17. Any important point highlighted in the research about measuring skin-carotenoid ………….
18. Study limitations ……………
